# Biochemical Characterization of Middle East Respiratory Syndrome Coronavirus Helicase

**DOI:** 10.1128/mSphere.00235-16

**Published:** 2016-09-07

**Authors:** Adeyemi O. Adedeji, Hilary Lazarus

**Affiliations:** Department of Pathology and Population Medicine, College of Veterinary Medicine, Midwestern University, Glendale, Arizona, USA; University of Maryland

**Keywords:** ATP hydrolysis, DNA, RNA, coronavirus, enzyme kinetics, helicase

## Abstract

Middle East respiratory syndrome coronavirus (MERS-CoV) helicase is a superfamily 1 helicase containing seven conserved motifs. We have cloned, expressed, and purified a Strep-fused recombinant MERS-CoV nonstructural protein 13 (M-nsp13) helicase. Characterization of its biochemical properties showed that it unwound DNA and RNA similarly to severe acute respiratory syndrome CoV nsp13 (S-nsp13) helicase. We showed that M-nsp13 unwound in a 5′-to-3′ direction and efficiently unwound the partially duplex RNA substrates with a long loading strand relative to those of the RNA substrates with a short or no loading strand. Moreover, the *K_m_* of ATP for M-nsp13 is inversely proportional to the length of the 5′ loading strand of the partially duplex RNA substrates. Finally, we also showed that the rate of unwinding (*ku*) of M-nsp13 is directly proportional to the length of the 5′ loading strand of the partially duplex RNA substrate. These results provide insights that enhance our understanding of the biochemical properties of M-nsp13.

**IMPORTANCE** Coronaviruses are known to cause a wide range of diseases in humans and animals. Middle East respiratory syndrome coronavirus (MERS-CoV) is a novel coronavirus discovered in 2012 and is responsible for acute respiratory syndrome in humans in the Middle East, Europe, North Africa, and the United States of America. Helicases are motor proteins that catalyze the processive separation of double-stranded nucleic acids into two single-stranded nucleic acids by utilizing the energy derived from ATP hydrolysis. MERS-CoV helicase is one of the most important viral replication enzymes of this coronavirus. Herein, we report the first bacterial expression, enzyme purification, and biochemical characterization of MERS-CoV helicase. The knowledge obtained from this study might be used to identify an inhibitor of MERS-CoV replication, and the helicase might be used as a therapeutic target.

## Abstract (precis)

Coronaviruses are known to cause a wide range of diseases in humans and animals. Middle East respiratory syndrome coronavirus (MERS-CoV) is a novel coronavirus discovered in 2012 and is responsible for acute respiratory syndrome in humans in the Middle East, Europe, North Africa, and the United States of America. Helicases are motor proteins that catalyze the processive separation of double-stranded nucleic acids into two single-stranded nucleic acids by utilizing the energy derived from ATP hydrolysis. MERS-CoV helicase is one of the most important viral replication enzymes of this coronavirus. Herein, we report the first bacterial expression, enzyme purification, and biochemical characterization of MERS-CoV helicase. The knowledge obtained from this study might be used to identify an inhibitor of MERS-CoV replication, and the helicase might be used as a therapeutic target.

## INTRODUCTION

Middle East respiratory syndrome coronavirus (MERS-CoV) is a novel coronavirus discovered in 2012 and is responsible for acute respiratory syndrome in humans in the Middle East (Saudi Arabia, Jordan, Qatar, and the United Arab Emirates), Europe (the United Kingdom, France, Italy, and Germany), North Africa (Tunisia and Egypt), and the United States of America (1, 2). Globally, as of 16 May 2016, the WHO has been notified of 1,733 laboratory-confirmed cases of infection with MERS-CoV, including at least 628 related deaths, in 27 different countries (http://www.who.int/csr/don/16-may-2016-mers-saudi-arabia/en/). While gastrointestinal symptoms, including diarrhea and queasiness, are also occasionally observed (3, 4), MERS-CoV causes mainly respiratory diseases.

MERS-CoV is a positive single-stranded RNA (ssRNA) virus with one of the largest known RNA genomes (30.119 kb) (5). Following infection, there is translation of two large replicative polyproteins, pp1a (the ORF1a polyprotein) and pp1ab (the polyprotein made from ORF1a and ORF1b through a −1 ribosomal frameshift during translation). These polyproteins are processed by the virus-encoded papain-like proteinase (PLpro) and nonstructural protein 5 (nsp5), a 3C-like protease (3CLpro). This auto-proteolysis leads to the formation of 16 nonstructural proteins, including an RNA-dependent RNA polymerase (RdRp) and a nucleoside triphosphatase (NTPase) and helicase that are known as nsp12 and nsp13, respectively (3, 6–8). These are likely to form the core of membrane-bound replication-transcription complexes in double-membrane vesicles at perinuclear regions (9).

Helicases are motor proteins that catalyze the processive separation of double-stranded nucleic acids (ds) into two single-stranded nucleic acids (ss) by utilizing the energy derived from ATP hydrolysis (10–17). Helicases are known to unwind nucleic acids during replication, recombination, and DNA repair (14), and recent reports have shown that they are also involved in other biological processes, including displacement of proteins from nucleic acid, movement of Holliday junctions, chromatin remodeling, catalysis of nucleic acid conformational changes (18–23), and several aspects of RNA metabolism (transcription, mRNA splicing, mRNA export, translation, RNA stability) and mitochondrial gene expression (9). A more recent biochemical study revealed a dual function of human enterovirus nonstructural protein 2C^ATPase^ as both an RNA helicase and an ATP-independent RNA chaperone which may destabilize RNA duplexes and assist in the formation of more globally stable RNA structures (24). Defects in helicase function have been associated with some human diseases, including Bloom’s syndrome, Werner’s syndrome, and xeroderma pigmentosum (25–28).

Previously, biochemical characterization of severe acute respiratory syndrome CoV (SARS-CoV) helicase (S-nsp13) demonstrated that S-nsp13 can unwind both double-stranded DNA and RNA in a 5′-to-3′ direction, and it can hydrolyze all deoxyribonucleotide triphosphates (dNTPs) and ribonucleotide triphosphates (29, 30), Additional study also showed that nsp12, the SARS-CoV RdRp, enhances the catalytic efficiency of S-nsp13 by increasing the step size of nucleic acid (RNA/RNA or DNA/DNA) unwinding by 2-fold (31). However, there are no reports on the biochemical characterization of MERS-CoV helicase (M-nsp13).

Herein, we report the first bacterial expression, enzyme purification, and biochemical characterization of M-nsp13. With double-stranded RNA (dsRNA) as a substrate, M-nsp13 unwound the substrate in a 5′-to-3′ direction, with the helicase requiring more ATP for optimal unwinding of RNA substrates with short 5′ loading strands. We also report that the rate of unwinding (*ku*) of M-nsp13 is directly proportional to the length of the 5′ loading strand of the partially duplex RNA substrate. While M-nsp13 required a single-strand 5- to 20-nucleotide (nt) 5′ overhang for efficient unwinding of the dsRNA substrate, M-nsp13 was still able to partially unwind (~10% to 30%) a dsRNA substrate with a 0-nt (blunt-ended) to 2-nt 5′ loading strand.

## RESULTS

### Expression and purification of MERS-CoV helicase (M-nsp13).

**FIG 1:**
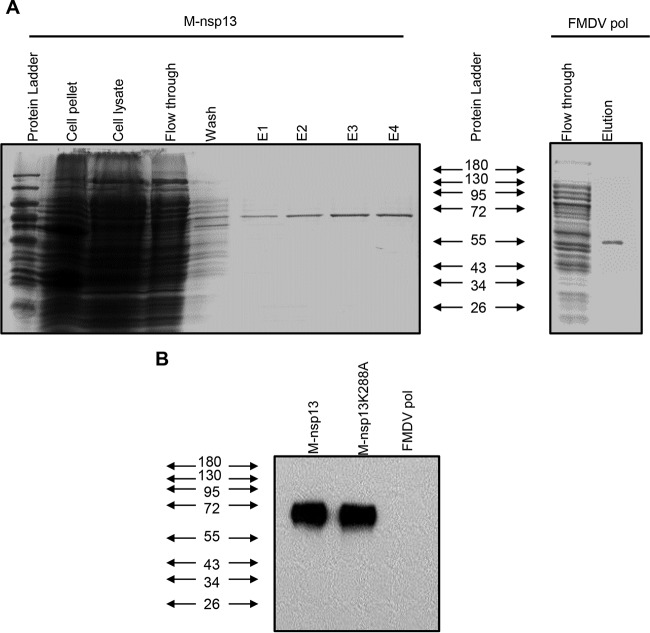
Protein expression and purification. (A) M-nsp13 and FMDV polymerase (pol) genes were cloned in pET52b and pet-28b, respectively, followed by their expression in the *E. coli* BL21 bacterial expression system. M-nsp13 was then purified by Strep-Tactin affinity chromatography. The FMDV polymerase was purified using nickel affinity chromatography. Purified M-nsp13 and FMDV polymerase were readily visualized by Coomassie blue staining of SDS-PAGE gels as ~68-kDa and 55-kDa protein products, respectively, in line with their expected molecular masses. (B) Western immunoblot analysis with M-nsp13-specific rabbit antiserum. The positions of protein molecular mass markers are indicated on the left (in kilodaltons).

**FIG 2:**
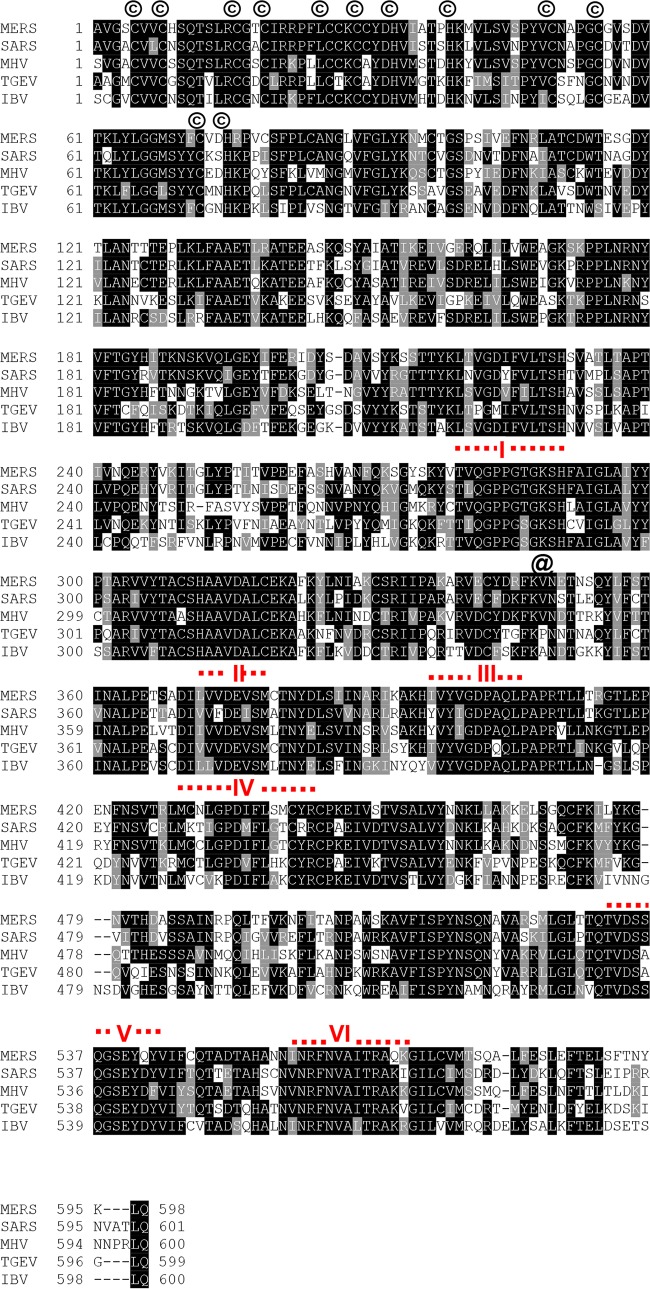
Sequence comparison of coronavirus helicases. The alignment was generated with the UniProt program (http://www.uniprot.org/align/) and the nsp13 sequences of MERS-CoV (human betacoronavirus strain 2c EMC/2012; GenBank accession no. JX869059.2), SARS-CoV (isolate Frankfurt 1; accession no. AY291315), mouse hepatitis virus (MHV; strain A59; accession number NC_001846), transmissible gastroenteritis virus (TGEV; strain Purdue 46; accession number AJ271965), and avian infectious bronchitis virus (IBV; strain Beaudette; accession number M95169) were derived from the replicative polyproteins of these viruses, whose sequences were obtained from the GenBank database. Conserved helicase motifs I to VI are indicated. Also indicated by an @ sign is the conserved Lys288 residue (corresponding to Lys5598 in pp1ab), which in the M-nsp13_K288A control protein was replaced with Ala. Lys288 is part of a conserved helicase motif (I) which is also called the Walker A box. Near the N terminus, the 12 conserved Cys and His residues predicted to form a binuclear zinc-binding cluster are indicated by a circled “C.” Overall, MERS helicase has 72.4% identity with SARS, 67.2% identity with MHV, 61.3% identity with TGEV, and 59.1% with IBV helicases.

To obtain sufficient amounts of M-nsp13 for biochemical studies, an *Escherichia coli* expression system was used. MERS-CoV pp1ab residues 5311 to 5881 were fused at the N terminus to the *E. coli* Strep tag. As Fig. 1A shows (lanes E1 to E4), fusion protein Strep–M-nsp13 of sufficient purity was obtained with a two-step purification protocol involving Strep-Tactin affinity and size exclusion chromatography. The same method was used to express and purify a Strep–M-nsp13 control protein, M-nsp13_K288A, in which the conserved lysine residue (5598 in pp1ab) of the Walker A box (32) (helicase motif I in Fig. 2) was replaced with Ala. The identities of the proteins were confirmed by Western blotting (Fig. 1B).

The amino acid sequence of M-nsp13 was aligned with the helicase amino acid sequences of other coronaviruses, including SARS-CoV isolate Frankfurt 1 (GenBank accession no. AY291315), mouse hepatitis virus (MHV) strain A59 (accession number NC_001846), porcine transmissible gastroenteritis virus (TGEV) strain Purdue 46 (accession number AJ271965), and avian infectious bronchitis virus (IBV) strain Beaudette (accession number M95169). The protein alignment shows that all helicases, along with M-nsp13, have the conserved helicase motifs I to VI, with motif I (Walker A box) containing the Lys288 that is recognized as the NTP binding site and the core ATPase site of superfamily 1 to 3 (SF1 to -3) helicases (32).

### M-nsp13 can unwind both RNA and DNA helices.

**FIG 3:**
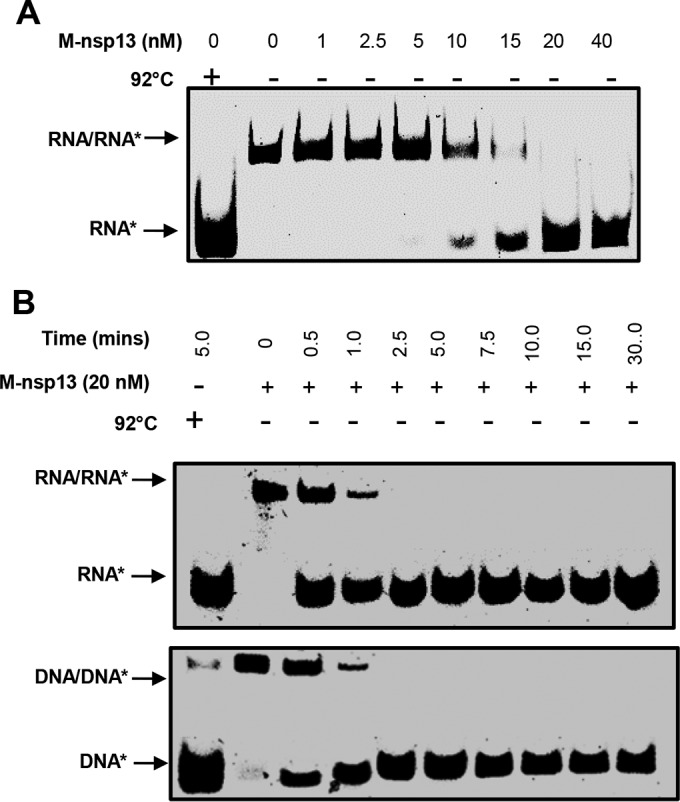
Helicase activity of M-nsp13. (A) Determination of optimal enzyme concentration. The activity of purified M-nsp13 was determined in an enzyme-dependent manner using a 5′-Cy3-labeled (*) partially duplex 5′-RNA-20 (20 ss, 22 ds) RNA substrate for 30 min. The reaction products were separated on a nondenaturing 8% polyacrylamide gel and visualized using the Bio-Rad multipurpose imager. (B) Comparison of fractions of partially duplex RNA and DNA substrates unwound by M-nsp13. The activity of purified M-nsp13 was verified in a time-dependent manner using a 5′-Cy3-labeled (*) partially duplex 5′-RNA-20 (20 ss, 22 ds) RNA substrate (upper gel) and a 5′-DNA-20 (20 ss, 22 ds) DNA substrate (lower gel). The reaction products were separated on a nondenaturing 8% polyacrylamide gel and visualized using the Bio-Rad multipurpose imager.

To evaluate the RNA helix unwinding activity of M-nsp13, various concentrations of the enzymes (1 to 40 nM) were incubated with a 5 nM concentration of the partially duplex RNA substrate with a 20-nt overhang (5′-RNA-20 [20 ss, 22 ds]) for 30 min. Unless stated otherwise, for this specific experiment, we used the 5′-RNA-20 partially duplex substrate (see Fig. S1 in the supplemental material), since previous coronavirus helicase, i.e., SARS-CoV nsp13, was shown to have a 5′-to-3′ directionality (29). As shown in Fig. 3A, the substrate was completely unwound at a 20 nM enzyme concentration in a 5′-to-3′ direction.

10.1128/mSphere.00235-16.1 Figure S1  Oligonucleotides and substrates used in this study. The Cy3-labeled strands are marked by asterisks. The green sequences denote the complementary sequences in the two strands. Download Figure S1, PDF file, 0.1 MB. Copyright © 2016 Adedeji and Lazarus. 2016 Adedeji and Lazarus This content is distributed under the terms of the Creative Commons Attribution 4.0 International license.

Some reports have shown that some helicases have a clear preference for either RNA or DNA (9, 33–36). To determine whether M-nsp13 prefers one substrate over the other, we used the 5′-overhang 20-nt (20 ss, 22 ds) RNA and DNA substrates (Fig. S1) and monitored M-nsp13’s helicase activity. As shown in Fig. 3B, M-nsp13 did not exhibit any preference for either RNA or DNA substrates. The results are similar to what was previously reported for SARS-CoV helicase (29). As such, our experimental analyses were carried out using RNA substrates to mimic the natural substrate.

### Metal and pH dependence of M-nsp13.

**FIG 4:**
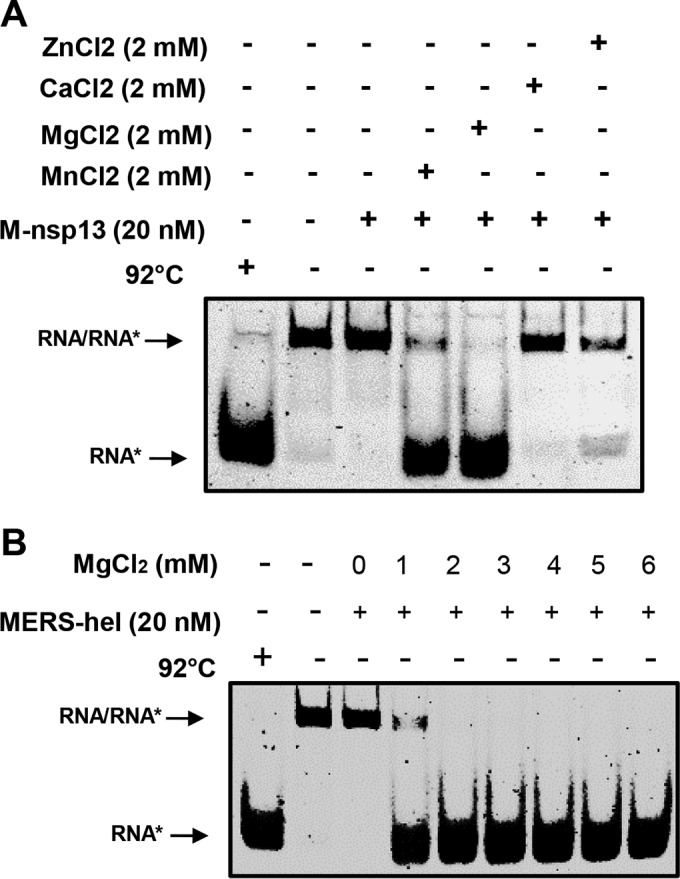
Determination of metal requirement. (A) The activity of purified M-nsp13 was performed using a 5′-Cy3-labeled (*) partially duplex 5′-RNA-20 (20 ss, 22 ds) RNA substrate in the presence of 2 mM of Zn^2+^, Ca^2+^, Mg^2+^, and Mn^2+^. The reaction products were separated on a nondenaturing 8% polyacrylamide gel and visualized using the Bio-Rad multipurpose imager. (B) Purified M-nsp13 was incubated with the 5′-Cy3-labeled (*) partially duplex 5′-RNA-20 (20 ss, 22 ds) RNA substrate in the presence of various concentrations of Mg. The reaction products were separated on a nondenaturing 8% polyacrylamide gel and visualized using the Bio-Rad multipurpose imager. hel, helicase.

To determine the metal requirements of M-nsp13 for its helicase activity, a 20 nM enzyme concentration was incubated with the 5′-RNA-20 (20 ss, 22 ds) substrate in the presence of 2 mM MnCl_2_, MgCl_2_, ZnCl_2_, or CaCl_2_. The results showed that M-nsp13 had the best helicase activity with MgCl_2_, followed by MnCl_2_, with more than 85% of the substrate unwound. The enzyme showed lesser activity with ZnCl_2_ and no observable activity in the presence of CaCl_2_ (Fig. 4A).

To determine the optimal concentration of MgCl_2_ required for M-nsp13 activity, various concentrations of MgCl_2_ (1 to 6 mM) were incubated with 20 nM M-nsp13 and the 5 nM 5′-RNA-20 (20 ss, 22 ds) substrate for 30 min. The result showed that M-nsp13 had optimal activity with 2 mM MgCl_2_ (Fig. 4B).

**FIG 5:**
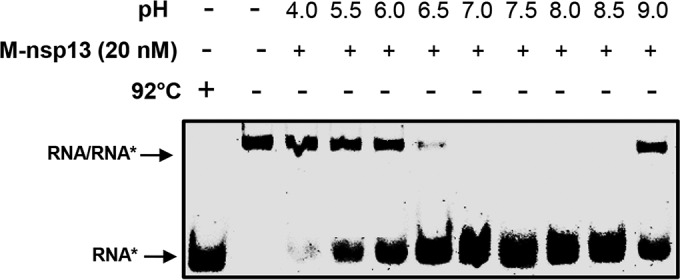
pH dependence. Purified M-nsp13 was incubated with the 5′-Cy3-labeled (*) partially duplex 5′-RNA-20 (20 ss, 22 ds) RNA substrate in reaction buffers with different pHs (4.0 to 9.0). The reaction products were separated on a nondenaturing 8% polyacrylamide gel and visualized using the Bio-Rad multipurpose imager.

To evaluate the optimal pH for M-nsp13 unwinding activity, reactions similar to those described above were performed at various pHs (4.0 to 9.0). As shown in Fig. 5, M-nsp13 has optimal unwinding activity from pHs 7.0 to 8.5.

### Minimum 5′ loading strand length requirement of M-nsp13 for efficient RNA unwinding.

**FIG 6:**
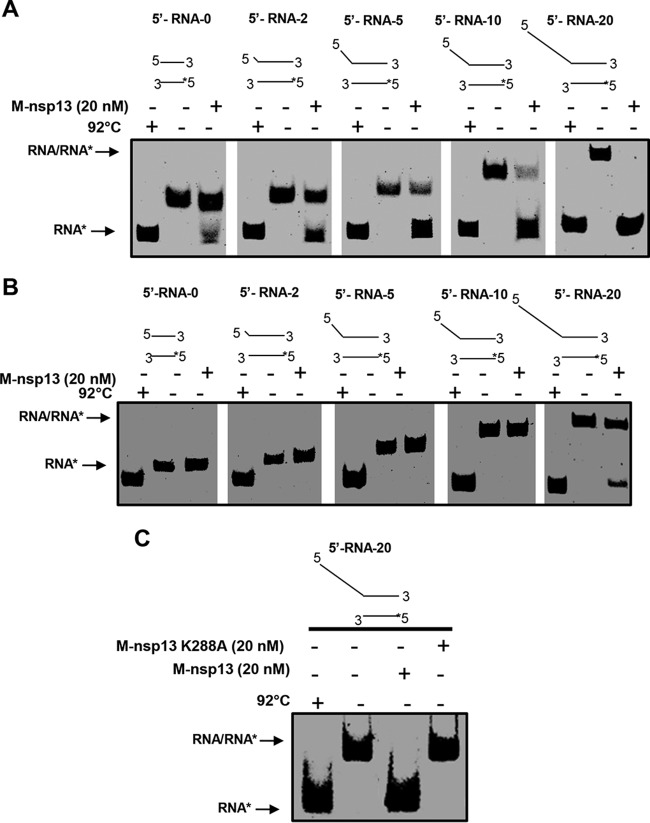
Substrate specificity, minimum overhang length, and ATP requirement for M-nsp13. (A and B). Five different substrates with 5′-overhang lengths varying from 0 to 20 nucleotides were designed to determine the minimum length of the loading strand required by M-nsp13 to efficiently unwind its substrate. The helicase activity of M-nsp13 (20 nM) was assessed on these substrates (5 nM each) at 30°C with 2 mM ATP (A) and no ATP (B). The products were separated on a nondenaturing 8% polyacrylamide gel and visualized using the Bio-Rad multipurpose imager. (C) Purified M-nsp13 or M-nsp13_K288A (20 nM) was incubated with the 5′-Cy3-labeled (*) partially duplex 5′-RNA-20 (20 ss, 22 ds) RNA substrate (5 nM) in the presence of 2 mM ATP. The reaction products were separated on a nondenaturing 8% polyacrylamide gel and visualized using the Bio-Rad multipurpose imager.

To determine whether nucleic acid unwinding by M-nsp13 depends on the loading strand length and to determine the minimum length of the 5′ single-stranded overhang required for efficient helicase activity, we designed five partially duplex RNA substrates with single-stranded 5′ ends of various lengths (between 0 and 20 bases) (see Fig. S1 in the supplemental material; Fig. 6) but with dsRNA duplex regions of the same length. Using these substrates, we monitored the unwinding activity of M-nsp13 in the presence of 2 mM ATP. As shown in Fig. 6A, the efficiency of RNA unwinding by M-nsp13 increased as the length of the 5′ loading strand increased. While M-nsp13 required a 5- to 20-nucleotide (nt) single-strand 5′ overhang for efficient unwinding of the dsRNA substrate, M-nsp13 was still able to partially unwind (~10% to 30%) a dsRNA substrate with a 0- (blunt) to 2-nt 5′ loading strand.

### The optimal helix unwinding activity of M-nsp13 requires NTPs.

NTP hydrolysis is known to provide the energy required for translocation of RNA helicases along ssRNA and duplex-RNA during unwinding. To determine whether ATP is required for the unwinding activities of M-nsp13, a standard unwinding assay was conducted with all the RNA substrates described above in the absence of ATP. As shown in Fig. 6B, while M-nsp13 was partially able to unwind the partially duplex RNA substrate with a 20-nt overhang (5′-RNA-20) in the absence of ATP, M-nsp13 could not unwind other RNA substrates with shorter loading strands.

A recently identified human enterovirus helicase, nonstructural protein 2C^ATPase^, was shown to possess an ATP-independent RNA chaperone activity (24). To investigate whether M-nsp13 has an underlying ability to unwind its substrate in an ATP-independent manner, M-nsp13_K288A (the control protein that lacks the ability to hydrolyze ATP) was incubated with the 5′-RNA-20 partially duplex substrate in the presence of 2 mM ATP. The results showed that M-nsp13_K288A could not unwind the 5′-RNA-20 partially duplex substrate, indicating that M-nsp13 does not have an ATP-independent RNA chaperone activity (Fig. 6C). Overall, these results suggest that M-nsp13’s ability to unwind the 5′-RNA-20 partially duplex substrate is likely due to residual cellular ATP purified with the helicase as well as to a long overhang length, which enhances M-nsp13’s unwinding activity.

**FIG 7:**
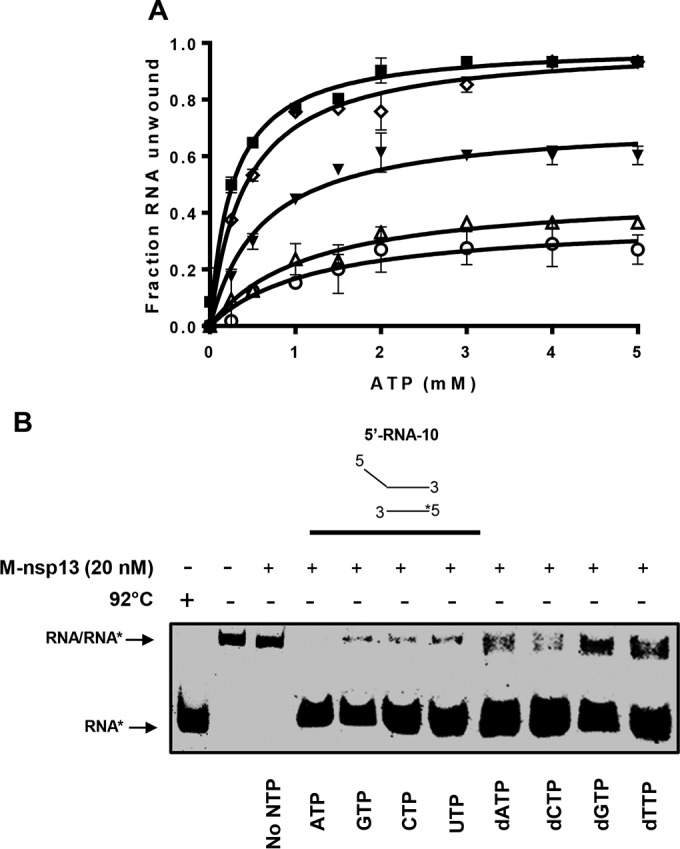
(d)NTP requirements for MERS helicase-unwinding activities. (A) Five different 5′-Cy3-labeled (*) partially duplex RNA substrates with 5′-overhang lengths varying from 0 to 20 nucleotides, i.e., 0 ss, 22 ds (○), 2 ss, 22 ds (Δ), 5 ss, 22 ds (▼), 10 ss, 22 ds (◇), and 20 ss, 22 ds (■) RNA substrates, were reacted with M-nsp13 (20 nM) in the presence or absence of increasing concentrations (0 to 5 mM) of ATP for 30 min. The reaction products were separated on a nondenaturing 8% polyacrylamide gel and visualized using the Bio-Rad multipurpose imager. The unwinding activities under different ATP concentrations were plotted as the fraction of the RNA released from the total RNA helix substrate (*y* axis) at each ATP concentration (*x* axis) using the Michaelis-Menten equation. The values for *K_m_* and *V*_max_ are provided in Table 1. Error bars represent standard deviation (SD) values from two separate experiments. (B) The 5′-Cy3-labeled (*) partially duplex 5′-RNA-10 (10 ss, 22 ds) RNA substrate (5 nM) was reacted with M-nsp13 (20 nM) in the presence or absence of the indicated NTPs (2 mM) for 30 min. The reaction products were separated on a nondenaturing 8% polyacrylamide gel and visualized using the Bio-Rad multipurpose imager.

Given the observation that M-nsp13 was partially able to unwind the 5′-RNA-20 partially duplex substrate when ATP was not included in the reaction mixture, we sought to investigate the ATP requirement of M-nsp13 for the different RNA substrates with 5′ loading strands of various lengths. We assessed the unwinding activity of M-nsp13 with the various 5′-RNA partially duplex substrates with different overhang lengths in the presence of various ATP concentrations from 0.25 to 5 mM. Using the Michaelis-Menten equation, the unwinding activities under different ATP concentrations were plotted as the fraction of the released RNA from the total RNA helix substrate at each ATP concentration. The results showed that increasing ATP concentrations up to ~2 mM ATP apparently enhanced the helix unwinding of each RNA substrate (Fig. 7A), after which no significant difference in substrate unwinding was noticeable. Moreover, M-nsp13 could not completely unwind the 5′-RNA-0 (blunt substrate), 5′-RNA-2 (2-nt overhang), and 5′-RNA-5 (5-nt overhang) partially duplex RNA substrates even at 5 mM ATP. Furthermore, the *K_m_* and *V*_max_ values for each substrate (Table 1) indicate that the amount of ATP required by M-nsp13 to unwind the substrates is inversely proportional to the length of the loading strand.

**TABLE 1:** Kinetic parameters for the ATP hydrolysis of M-nsp13 with partially duplex RNA substrates with loading strands of various lengths^a^

RNA substrate	*V*_max_ (min^−1^)	*K_m_* (ATP) (mM)
5′-RNA-0 (0 ss, 22 ds)	0.37 ± 0.05	1.23 ± 0.49
5′-RNA-2 (2 ss, 22 ds	0.47 ± 0.04	1.14 ± 0.28
5′-RNA-5 (5 ss, 22 ds)	0.72 ± 0.03	0.62 ± 0.09
5′-RNA-10 (10 ss, 22 ds)	0.99 ± 0.02	0.41 ± 0.04
5′-RNA-20 (20 ss, 22 ds)	0.99 ± 0.02	0.26 ± 0.03

a The *V*_max_ and *K_m_* values were determined by fitting the data by nonlinear regression to the Michaelis-Menten equation using Prism 7.0 software. Experiments were performed two times.

To further investigate the (d)NTP requirements of M-nsp13, a standard unwinding assay was conducted as described above, using the 5′-RNA-10 (10 ss, 22 ds [or 10-nt overhang]) partially duplex RNA substrate in the presence or absence of individual (d)NTPs (2 mM). The results showed that M-nsp13 was able to hydrolyze all the nucleotides for the purpose of nucleic acid unwinding, with slightly more specificity for the NTPs (Fig. 7B). These results further confirmed that the helix unwinding activity of M-nsp13 needs the participation of ATP or other NTPs. It should be noted that since M-nsp13 can partially unwind the 5′-RNA-20 (20 ss, 22 ds [or 20-nt overhang]) partially duplex RNA substrate in the absence of ATP, we decided not to use this substrate for this experiment to allow for proper assessment of the effect of NTPs on M-nsp13’s unwinding activity.

### M-nsp13 rates of unwinding of the partially duplex RNA substrates that have various 5′ overhang lengths.

**FIG 8:**
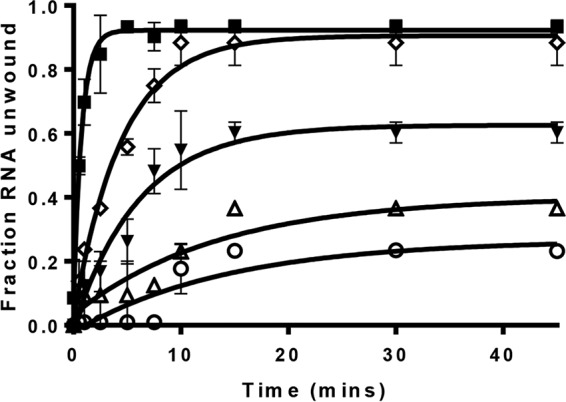
M-nsp13 rates of unwinding of all RNA substrates with 5′ loading strands of various lengths. (A) Five different 5′-Cy3-labeled (*) partially duplex RNA substrates with 5′-overhang lengths varying from 0 to 20 nucleotides, i.e., 0 ss, 22 ds (○), 2 ss, 22 ds (Δ), 5 ss, 22 ds (▼), 10 ss, 22 ds (◇), and 20 ss, 22 ds (■), were reacted with M-nsp13 (20 nM) in the presence of 2 mM ATP for 0 to 45 min. The reaction products were separated on a nondenaturing 8% polyacrylamide gel and visualized using the Bio-Rad multipurpose imager. The derived fractions of RNA unwound at the indicated time points were plotted against time (in minutes), and the data points were fit to a single-exponential equation (see Results) to determine the rates of unwinding (*ku*) of all substrates (Table 2). Error bars represent SD from two separate experiments.

To determine the rates of unwinding of M-nsp13 with all the partially duplex RNA substrates with various 5′ overhang lengths used in this study, the enzyme was incubated with the RNA substrates 5′-RNA-0 (the blunt substrate), 5′-RNA-2 (2-nt overhang), 5′-RNA-5 (5-nt overhang), 5′-RNA-10 (10-nt overhang), and 5′-RNA-20 (20-nt overhang) in the presence of 2 mM ATP at different time points (0 to 45 min). The change in the fractions of unwound ssRNA product with time is shown in Fig. 8. The experimental data for the substrates were fit to the following equation: fraction (ssDNA) = *A* ⋅ (1 – *e^–kt^*) + *n*, where *A* is the amplitude that corresponds to the maximum fraction of ssRNA that can be generated enzymatically from the substrates, *k* is the pseudo first-order rate constant of RNA unwinding, *t* is the reaction time, and *n* is an additive constant representing the amount of ssDNA present before the reaction started. The results showed that the amplitudes and the rates of RNA unwinding of M-nsp13 were directly proportional to the length of the 5′ loading strand (Table 2).

**TABLE 2:** Kinetic parameters for M-nsp13 rates of unwinding and amplitudes for the different partially duplex RNA substrates with 5′ overhangs of various lengths^a^

RNA substrate	Amplitude	*ku* (s^−1^)
5′-RNA-0 (0 ss, 22 ds)	0.27 ± 0.04	0.07 ± 0.03
5′-RNA-2 (2 ss, 22 ds)	0.40 ± 0.06	0.08 ± 0.03
5′-RNA-5 (5 ss, 22 ds)	0.63 ± 0.03	0.17 ± 0.03
5′-RNA-10 (10 ss, 22 ds)	0.90 ± 0.02	0.42 ± 0.02
5′-RNA-20 (20 ss, 22 ds)	0.92 ± 0.02	1.29 ± 0.14

a The rates of RNA unwinding (*ku*) and amplitudes were determined by fitting the data by means of nonlinear regression to a single-exponent equation (see Results) using Prism 7.0 software. Experiments were performed two times.

### M-nsp13 is a unidirectional helicase.

**FIG 9:**
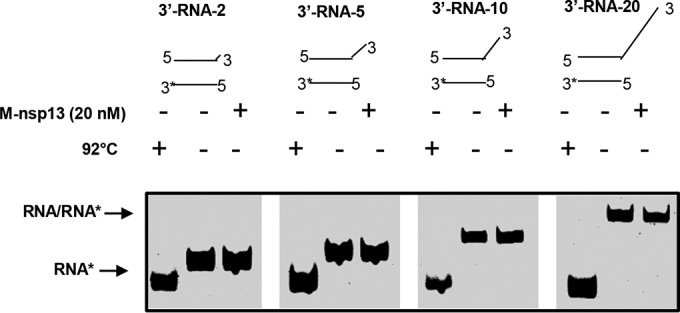
M-nsp13 does not unwind partially duplex RNA substrates with a 3′ loading strand. Four different substrates with 3′-overhang lengths varying from 2 to 20 nucleotides were designed to determine whether M-nsp13 can unwind in a 3′-to-5′ direction. The helicase activity of M-nsp13 (20 nM) was assessed on these substrates (5 nM each) at 30°C for 5 min in the presence of 2 mM ATP. The products were separated on a nondenaturing 8% polyacrylamide gel and visualized using the Bio-Rad multipurpose imager.

Previous reports have shown that SARS-CoV helicase has a 5′-to-3′ directionality and, as such, does not show any activity with a substrate that has a 3′ overhang (29, 31). To determine whether M-nsp13 can also unwind in a 3′-to-5 direction, the standard helicase assay was conducted with the RNA substrates that have various lengths of the 3′ loading strand in the presence of 2 mM ATP. As shown in Fig. 9, M-nsp13 could not unwind any of the 3′ RNA (3′-overhang) substrates, confirming that M-nsp13 is a unidirectional 5′-to-3′ helicase.

## DISCUSSION

Sequence alignment of M-nsp13 with other helicases suggests that the MERS-CoV helicase belongs to the SF1 family of helicases (29). However, the biochemical aspects of the enzymatic mechanism of MERS-CoV nsp13, including ATP hydrolysis, translocation along the nucleic acid, and the unwinding rate, have not been studied. The present study fills this knowledge gap.

One of the unique findings of this study is the observation that the length of the 5′ loading strand of the partially duplex RNA can determine the amount of ATP required by the helicase for optimal helix unwinding. The longer the length of the loading strand, the lower the amount of ATP required for optimal unwinding. As noted in Fig. 6B, M-nsp13 was able to partially unwind the substrate with the longest 5′ loading strand without inclusion of ATP in the reaction mixture. A recently characterized human enterovirus helicase, nonstructural protein 2C^ATPase^, was shown to possess an ATP-independent RNA chaperone activity (24). The RNA chaperone activity was observed along the 3′-to-5′ direction in the absence of ATP. Given the presence of an ssRNA product in the absence of ATP, when M-nsp13 was incubated with the 5′-RNA-20 partially duplex RNA substrate, we wanted to confirm a possible RNA chaperone activity by M-nsp13. As shown in Fig. 6C, M-nsp13_K288A (the control protein that lacks the ability to hydrolyze ATP) did not unwind either substrate, thereby ruling out an RNA chaperone activity by M-nsp13. The small amount of the ssRNA product observed with the 5′-RNA-20 partially duplex RNA substrate in the absence of ATP may be due to residual cellular ATP purified with the helicase as well as the longer overhang length of 5′-RNA-20 substrate relative to other substrates that enhances M-nsp13 unwinding activity.

Another important finding in this study is that the rate of unwinding of M-nsp13 is directly proportional to the length of the 5′ loading strand of the partially duplex RNA substrate. This observation may be due to the ease of binding the long loading strand by M-nsp13.

Previous reports have shown that SARS-CoV nsp12 (RNA polymerase) and nsp13 (helicase) physically interact, with nsp12 enhancing the activity of nsp13 (31, 37), thereby forming part of a larger SARS-CoV replication complex. The 5′-to-3′ helicase activity of M-nsp13 may be similar to what was previously proposed for SARS-CoV (31). It was suggested that SARS-CoV nsp12 and nsp13 may work together to carry out one of the replication complex functions, i.e., synthesis of subgenomic transcripts containing the leader sequence derived from the 5′ end of the genome (29, 30). To achieve this, nsp12 would have to transcribe RNA in a 3′-to-5′ template direction, followed by pausing at transcription regulatory element sites. Such pauses may activate motion in the opposite direction, allowing nsp13 (aided by nsp12 and possibly other viral and host proteins) to unwind the 3′ end of the nascent RNA primer and facilitate transfer to the complementary region of the 5′ leader genomic sequence.

Previous studies revealed that SARS-CoV and human coronavirus 229E helicases required at least a 5-nt 5′-end single-strand overhang for efficient unwinding (31, 38). In this study, while up to 10 to 30% of ssRNA products were observed with the 0- to 2-nt 5′-end single-strand overhang partially duplex RNA substrates, M-nsp13 exhibited efficient unwinding activities only with partially duplex RNA substrates that have a ≥5-nt-long 5′ loading strand.

While some helicases exhibit a clear preference for either RNA or DNA (9, 33–36), some helicases, such as SARS-CoV helicase, worked well with either RNA or DNA (31). Our findings with M-nsp13 also support the fact that coronavirus helicases do not exhibit a preference for either RNA or DNA substrates.

Comparison of the NTPase and dNTPase activities of M-nsp13 showed moderate variations, as M-nsp13 clearly exhibited better enzymatic activities with the NTPs than with the dNTPs. Nonetheless, the data suggested that the nucleotide-binding site of M-nsp13 has a low specificity among the NTPs. Similar data were also obtained for SARS-CoV nsp13 (30). Other RNA virus helicase-associated NTPase activities were also shown to lack any noticeable specificity for specific nucleotides (30, 35, 39–41). This may suggest that the lack of selectivity for specific nucleotide cofactors is a general feature of RNA virus helicases. Due to the high nucleotide consumption necessary for maximum viral RNA synthesis in the host cell, it may be beneficial for the helicase’s duplex-unwinding activity not to depend strictly on a specific nucleotide. As such, there is lower risk for depletion of cellular NTP pools.

Other areas for future investigation include establishing the kinetic step size and the natural state of the active protein, .i.e., the monomer of multimers, and characterizing replication complex functions that involve M-nsp13, along with other MERS-CoV nonstructural proteins.

In conclusion, we report the first expression, purification, and biochemical characterization of MERS-CoV helicase. We showed that M-nsp13 required more ATP for optimal unwinding of partially duplex RNA substrates with short 5′ loading strands. Our results also revealed that the rate of unwinding of M-nsp13 increased with a partially duplex RNA substrate with a longer 5′ loading strand. These results provide insights that enhance our understanding of the biochemical properties of M-nsp13. As such, M-nsp13 might be used as a target to identify inhibitors of MERS-CoV replication.

## MATERIALS AND METHODS

### Cloning, expression, and purification of M-nsp13 helicase.

M-nsp13 is encoded by nucleotides 16208 to 18001 of the MERS-CoV genome (human betacoronavirus strain 2c EMC/2012; GenBank accession no. JX869059.2). The coding region of M-nsp13 was amplified by PCR using 5′-BamHI-nsp13-CGGGATCCTGCTGTAGGCTCTTGTGTTG-3′ as the forward primer and 3′-EagI-nsp13-ATGCGGCCGCTATTGCAGCTTGTAGTTGGTAAAGCTC-3′ as the reverse primer. The PCR amplicons were digested with BamHI and EagI, followed by ligation into expression vector pET52b (Novagen-EMD Millipore), which fused M-nsp13 with a Strep tag at the N terminus. After confirmation of the amplicon nucleotide sequences, the construct was transformed into *E. coli* BL21 cells. Protein expression and purification were performed as described previously (1, 31, 42) using Strep-Tactin beads (IBA). The protein was eluted with 100 mM Tris, pH 8.0, 150 mM NaCl, 10 mM desthiobiotin, 0.1% Triton X-100, 5 mM β-mercaptoethanol (BME), and 5% glycerol. Using dialysis, the buffer was exchanged with (100 mM Tris, pH 8.0, 150 mM NaCl, 0.1% Triton X-100, 5 mM BME, and 5% glycerol). Fractions containing the desired protein were concentrated and stored at −80°C.

The M-nsp13_K288A mutant was prepared using the QuikChange II XL site-directed mutagenesis kit (Agilent) as described by the manufacturer and expressed and purified as described above for the wild-type enzyme.

### Expression and purification of foot-and-mouth disease virus (FMDV) 3Dpol.

Expression and purification were as previously described (43). Briefly, the pET-28a-FMDV 3Dpol plasmid was transformed into the Rosetta 2 expression strain (Novagen). Kanamycin-resistant colonies were grown at 37°C, and protein expression was induced at an *A*_600_ of 0.9 to 1.0 by the addition of 1 mM isopropyl β-d-1-thiogalactopyranoside (IPTG). The cells were allowed to grow for 3 more h after induction. The cells were harvested by centrifugation (4,500 × *g*, 20 min) and stored at −20°C. Frozen cell pellets were resuspended in buffer A (25 mM Tris-HCl, pH 8.0, 500 mM NaCl, and 5% glycerol). The protein was purified by nickel affinity chromatography with a gradient of 25 mM to 500 mM imidazole in buffer A. Fractions containing pure protein (95%) were pooled and dialyzed against the storage buffer containing 12.5 mM Tris-HCl, pH 8.0, 100 mM NaCl, and 50% glycerol.

### Western blotting.

Purified M-nsp13, M-nsp13K288A, and FMDV polymerase were separated by 10% SDS-polyacrylamide gel electrophoresis (PAGE), followed by transfer to nitrocellulose membranes (Schleicher & Schuell, Dassel, Germany). M-nsp13 was detected with anti-M-nsp13 rabbit antiserum as the primary antibody, which was commercially developed upon request (Abcam). A horseradish peroxidase-conjugated swine anti-rabbit immunoglobulin G (Dako) was used as the secondary antibody.

### Nucleic acid substrates.

Synthetic oligonucleotides were purchased from Integrated DNA Technologies (Coralville, IA). Sequences of the RNA and DNA substrates are shown in Fig. S1 in the supplemental material. Concentrations were determined spectrophotometrically using absorption at 260 nm and their extinction coefficients. Unlabeled oligonucleotides were annealed to corresponding 5′-Cy3-labeled 22-mers in 50 mM Tris, pH 8.0, 50 mM NaCl at a ratio of 1 to 1.2 by heating them at 95°C for 5 min and cooling them slowly to room temperature. Unlabeled 22-mers were used as traps for the helicase assay.

### Characterization of helicase activity.

Helicase activity was measured by incubating 20 nM M-nsp13 with a 5 nM concentration of an RNA substrate (Fig. S1) in a reaction buffer containing 20 mM HEPES, pH 7.5, 20 mM NaCl, 1 mM dithiothreitol (DTT), 0.1 mg/ml bovine serum albumin (BSA), 5 mM MgCl_2_, and 2 mM ATP at 30°C for various times. Reactions were quenched by the addition of an equal volume of loading buffer (0.2% SDS and 20% glycerol). Unless otherwise mentioned, reactant concentrations refer to the final concentration in the reaction mixture. The released single-stranded RNA (ssRNA) product and unwound double-stranded RNA (dsRNA) were resolved by 8% nondenaturing PAGE using a running buffer containing 89 mM Tris-borate, pH 8.2, and run for 45 min at 4°C and 110 V. The controls for measuring maximum unwinding were dsRNAs denatured by heat for 5 min at 95°C and loaded immediately on the gel. In this and subsequent assays, the gels were scanned with a Bio-Rad multipurpose imager. When necessary, the band intensities representing ssRNA and dsRNA were quantitated using the Image Lab 5.1 software (Bio-Rad). The fraction of unwound RNA was plotted against time, and the kinetic parameters described in the mechanism were determined by nonlinear regression using GraphPad Prism (GraphPad Inc.).

### Analyses of RNA unwinding.

To obtain the kinetic parameters associated with RNA unwinding by M-nsp13, the fraction of unwound RNA was plotted against time. By nonlinear regression, data were fit to the single-exponential equation in Results using Prism 7.0 (GraphPad Inc.).

To obtain the kinetic parameters associated with the *K_m_* and *V*_max_ of M-nsp13 with regard to ATP hydrolysis, the fraction of unwound RNA was plotted against different ATP concentrations. Data fitting was carried out by nonlinear regression to the Michaelis-Menten equation using Prism 7.0 (GraphPad Inc.).
