# Supplementary material for: Biochemical Characterization of Middle East Respiratory Syndrome Coronavirus Helicase
Source: mSphere. 2016 Sep 7;1(5):e00235-16. doi: 10.1128/mSphere.00235-16 (PMC5014916; doi:10.1128/mSphere.00235-16)
Supplement: Figure S1 [file sph005162143sf1.pdf]

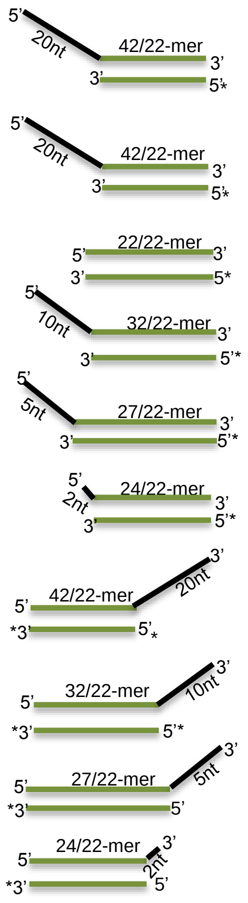

5'-TAGATAGCCATAGATAGCATTGGTGCTCGAACAGTGACTAGC-3'
3'-ACCACGAGCTTGTCACTGATCG-5'*

5'-UAGAUAGCCAUAGAUAGCAUUGGUGCUCGAACAGUGACUAGC-3'
3'-ACCACGAGCUUGUCACUGAUCG-5'*

5'-UGGUGCUCGAACAGUGACUAGC-3'
3'-ACCACGAGCUUGUCACUGAUCG-5'*

5'-UAGAUAGCAUUGGUGCUCGAACAGUGACUAGC-3'
3'-ACCACGAGCUUGUCACUGAUCG-5'*

5'-AGCAUUGGUGCUCGAACAGUGACUAGC-3'
3'-ACCACGAGCUUGUCACUGAUCG-5'*

5'-CCUGGUGCUCGAACAGUGACUAGC-3'
3'-ACCACGAGCUUGUCACUGAUCG-5'*

5'-UGGUGCUCGAACAGUGACUAGCUAGAUAGCCAUAGAUAGCAU-3'
*3'-ACCACGAGCUUGUCACUGAUCG-5'

5'-UGGUGCUCGAACAGUGACUAGCUAGAUAGCAU-3'
*3'-ACCACGAGCUUGUCACUGAUCG-5'

5'-UGGUGCUCGAACAGUGACUAGCAGCAU-3'
*3'-ACCACGAGCUUGUCACUGAUCG-5'

5'-UGGUGCUCGAACAGUGACUAGCCC-3'
*3'-ACCACGAGCUUGUCACUGAUCG-5'

Supplementary Figure 1. Oligonucleotides and substrates used in this study. The Cy3-labeled strands are marked by asterisks. The green sequences denote the complementary sequences in the two strands.
